# Preparation of cobalt based metal organic framework ZIF-67 catalyst for activating peroxymonosulfate and its catalytic system for rapid degradation of Rhodamine B and other dyes at high concentrations

**DOI:** 10.1039/d5ra08184j

**Published:** 2025-12-15

**Authors:** Yangyi Cai, Xiaocan Liu, Weiye Yang, Hongyan Peng, Lijian Meng, Shihua Zhao

**Affiliations:** a College of Physics and Electronic Engineering, Hainan Normal University Haikou 571158 China zsh@hainnu.edu.cn haha_weiye@163.com; b The Innovation Platform for Academicians of Hainan Province Haikou Hainan Province 571158 China; c CIETI/ISEP, Polytechnic of Porto Rua Dr. António Bernardino de Almeida 4249-015 Porto Portugal

## Abstract

Persistent organic pollutants pose significant long-term risks to environmental and human health due to their carcinogenic, mutagenic, and teratogenic properties. This study focuses on the efficient activation of peroxymonosulfate (PMS) through the rational design of a cobalt-based metal–organic framework (ZIF-67) catalyst, enabling rapid degradation of high-concentration dye pollutants. By optimizing the molar ratio of 2-methylimidazole (2-MeIM) to Co(NO_3_)_2_·6H_2_O and employing a hydrothermal synthesis method, we fabricated ZIF-67 (4 : 1) with high crystallinity, large specific surface area (1656.68 m^2^ g^−1^), and stable degradation performance. The ZIF-67 (4 : 1)/PMS system achieved complete degradation of 20 mg L^−1^ and 100 mg per L Rhodamine B (RhB) within 60 seconds and 6 minutes, respectively, surpassing recent reported efficiencies. The system also maintained high activity over a broad pH range (3–9). Radical quenching experiments and electron paramagnetic resonance (EPR) analysis identified sulfate radicals (˙SO_4_^−^_4_) and singlet oxygen (^1^O_2_) as the dominant reactive species. Furthermore, the catalyst exhibited excellent recyclability with no significant loss in activity after five consecutive cycles. This work provides a facile and scalable strategy for preparing highly active cobalt-based catalysts, demonstrating great potential for practical applications in industrial wastewater treatment and environmental remediation.

## Abstract 2

The mechanism of ZIF-67 catalyst for activating peroxymonosulfate to degrade RhB.

## Graphical abstract

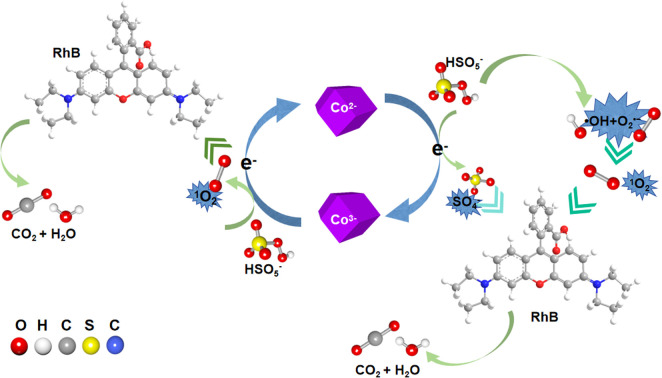

## 1. Introduction

Persistent organic pollutants remain in the environment for extended durations following their discharge.^1^ Owing to their toxic, carcinogenic, mutagenic, and teratogenic properties, even at low concentrations, their presence in wastewater may pose significant threats to both human health and aquatic organisms.^2^ As a class of emerging contaminants, dye molecules present substantial degradation challenges due to their widespread industrial utilaization and the large volumes of dye-containing wastewater released.^3,4^ The stability and xenobiotic nature of reactive dyes render their complete removal by conventional wastewater treatment processes challenging.^5–7^ Currently, the primary methods for treating dye wastewater include adsorption, membrane separation, oxidative degradation, and aerobic and anaerobic microbial degradation. However, each of these methods has inherent limitations. For instance, adsorption merely transfers pollutants from the aqueous phase to the adsorbent rather than achieving complete degradation; additionally, the adsorbent is prone to saturation and requires regeneration or disposal.^8^ Although membrane separation technology offers high separation efficiency, it confronts challenges such as membrane fouling, high operational costs, and the need for further treatment of the resulting concentrate.^9^ Conventional oxidative degradation methods, such as those utilizing chlorine or ozone, are sometimes inefficient and may produce toxic by-products.^10^ While microbial degradation is a viable approach, it often demands extended treatment periods, exhibits limited effecacy against recalcitrant dyes, and is highly sensitive to environmental factors such as pH, temperature, and toxicity.^11^ To address these limitations, advanced oxidation processes based on activated peroxymonosulfate (PMS) have emerged as a promising strategy for the non-selective degradation of persistent organic pollutants.^12–14^

The activation of peroxymonosulfate (PMS) is particularly well-suited for addressing persistent pollutants, encompassing a range of refractory organic contaminants such as dyes, antibiotics, and bacteria. This advanced technology generates highly reactive species (*e.g.* sulfate radicals SO_4_˙^−^ and hydroxyl radicals ˙OH), thereby enabling highly efficient and thorough mineralization of target pollutants. To date, PMS-based advanced oxidation processes have garnered significant advancements in research endeavors worldwide. For instance, Li Yanfang's team employed pyrolyzed activated carbon particles modified with trace ZIF-67 (cal-ZIF-67/AC) to activate PMS, which achieved a 98.2% degradation rate of 60 mg per L Rhodamine B (RhB) within 60 minutes, thus exhibiting excellent catalytic performance;^15^ the research team led by Kai Jiang fabricated a ZIF-67 hybrid network mediated *via* cellulose nanocrystals (ZIF-67@CNC) for peroxymonosulfate (PMS) activation, achieving a 98% degradation rate of 20 mg L^−1^ initial concentration Rhodamine B (RhB) within 5 minutes;^16^ the research team led by Fu Yang adopted a polymer-confined strategy for the modification of a ZIF-67-derived cobalt-confined nanocage (Co-NC@Co/C-6) in PMS activation, which achieved a 97.7% degradation rate of 20 mg L^−1^ initial concentration norfloxacin (NFX) within 20 minutes.^17^ The research team led by Sun Zhirong synthesized a catalyst (BC–Co–W-700) *via in situ* loading of cobalt tungstate and cobalt onto walnut shell biochar for PMS activation and this system achieved complete degradation of 20 mg L^−1^ initial concentration chlortetracycline (CTC) within 18 minutes.^18^ Although these studies highlight the substantial potential of cobalt-based catalysts—especially ZIF-67 and its derivatives—for PMS activation and subsequent pollutant degradation, notable limitations persist. These include a narrow pH operating range, inadequate stability and reusability, as well as a significant decline in degradation efficiency during the treatment of high-concentration pollutants.

To mitigate the aforementioned challenges, this study centers on the precise design and performance tuning of pristine ZIF-67 (a cobalt-based metal–organic framework) catalysts. ZIF-67, a zeolitic imidazolate framework-67, is a porous crystalline material constructed *via* the coordinated self-assembly cobalt ions and 2-methylimidazole ligands. It has found extensive applications in diverse fields spanning gas storage/adsorption,^19–26^ separation,^27–29^ catalysis,^30–39^ and dye/toxicant removal.^40–46^ ZIF-67 possesses exceptional porosity, chemical stability, and prominent ligand-to-metal charge-transfer characteristics.^47–50^ However, ZIF-67 catalysts are also plagued by inherent limitations, such as limited aqueous stability and a propensity for inducing secondary pollution, which severely impedes their practical application.

This study presents a facile and scalable synthesis approach for the fabrication of more stable ZIF-67 as a heterogeneous catalyst in the activation of PMS for dye removal. A simplified hydrothermal synthesis protocol was adopted, wherein the core emphasis lies on the systematic modulation of the molar ratio between 2-MeIM(2-methylimidazole) and Co(NO_3_)_2_·6H_2_O (*i.e.*, 4 : 1, 10 : 1, 20 : 1). This approach is designed to boost crystallinity and structural stability by leveraging the hydrothermal environment to promote atomic orderly arrangement, reduce defects, and improve the framework's resistance to aqueous/acidic conditions, ultimately enhancing cycling stability and reducing metal leaching.^51–53^ Elevated reaction temperatures promote ligand coordination, thus minimizing unreacted ligands which might otherwise block active sites. This optimization achieves an optimal balance between the accessibility of active sites (Co^2+^) and ligand coverage, simultaneously optimizing the material's specific surface area and pore structure—with a specific focus on tuning the mesopore fraction. These structural and textural enhancements facilitate PMS adsorption, pollutant diffusion, and the rapid generation and subsequent action of reactive oxygen species (ROS).

Comprehensive characterization results reveal that the ZIF-67 nanocrystals synthesized through the hydrothermal method possess excellent crystallinity and a large specific surface area. When employed as a catalyst in sulfate radical-based advanced oxidation processes (SR-AOPs) for degrading Rhodamine B (RhB), ZIF-67 exhibits outstanding catalytic activity. It enables ultra-fast and complete degradation of various dyes, including high concentrations (*e.g.* 100 mg L^−1^) of RhB, within minutes, while retaining excellent performance across a wide pH spectrum (3–9). Additionally, density functional theory (DFT) calculations demonstrate that the HOMO–LUMO gaps for the spin-up and spin-down states of ZIF-67 are 0.003 eV and 0.0173 eV, respectively. These narrow band gaps indicate that a low electron transfer energy barrier, thereby facilitating the Co^2+^/Co^3+^ redox cycle. This result highlights the exceptional electron transport capability of ZIF-67 and lays a theoretical basis for its efficient redox cycling during PMS activation.

This work presents a facile, efficient, and scalable strategy for the development of cobalt-based catalysts with outstanding catalytic performance. Through the integration of ligand ratio tuning and hydrothermal synthesis, we successfully synthesized a pristine ZIF-67 catalyst with high performance, excellent stability, and facile recyclability. This catalyst facilitates the establishment of a highly effective ZIF-67/PMS catalytic oxidation system, effectively addressing critical challenges associated with the treatment of high-concentration dye wastewater across a wide pH spectrum—such as degradation rate, efficiency, stability, and general applicability—thus offering more reliable technical support for practical applications. Moreover, through the elucidation of the distinct PMS activation mechanism, this study lays a robust theoretical and technical groundwork for the design of efficient, stable, and versatile pollutant degradation technologies customized for complex industrial wastewater environments, including those in chemical, dyeing, and pharmaceutical industries. The findings exhibit substantial potential for practical real-world implementation and are anticipated to drive the advancement of advanced wastewater treatment technologies.

## 2. Experimental materials and methods

### 2.1. Experimental materials

Chemical reagents, including Rhodamine B (RhB), methyl orange (MO), methylene blue (MB), ethanol, methanol (MeOH), NaOH, HCl, 2-methylimidazole (2-MeIM),cobalt nitrate hexahydrate (Co(NO_3_)_2_·6H_2_O), *tert*-butanol (TBA), *p*-benzoquinone (*p*-BQ), l-histidine (l-His), and peroxymonosulfate (PMS, ≥42% KHSO_5_ basis), were purchased from Aladdin Ltd (Shanghai, China). All chemicals were of analytical grade and used without further purification.

### 2.2. Catalyst preparation

**Scheme 1:**
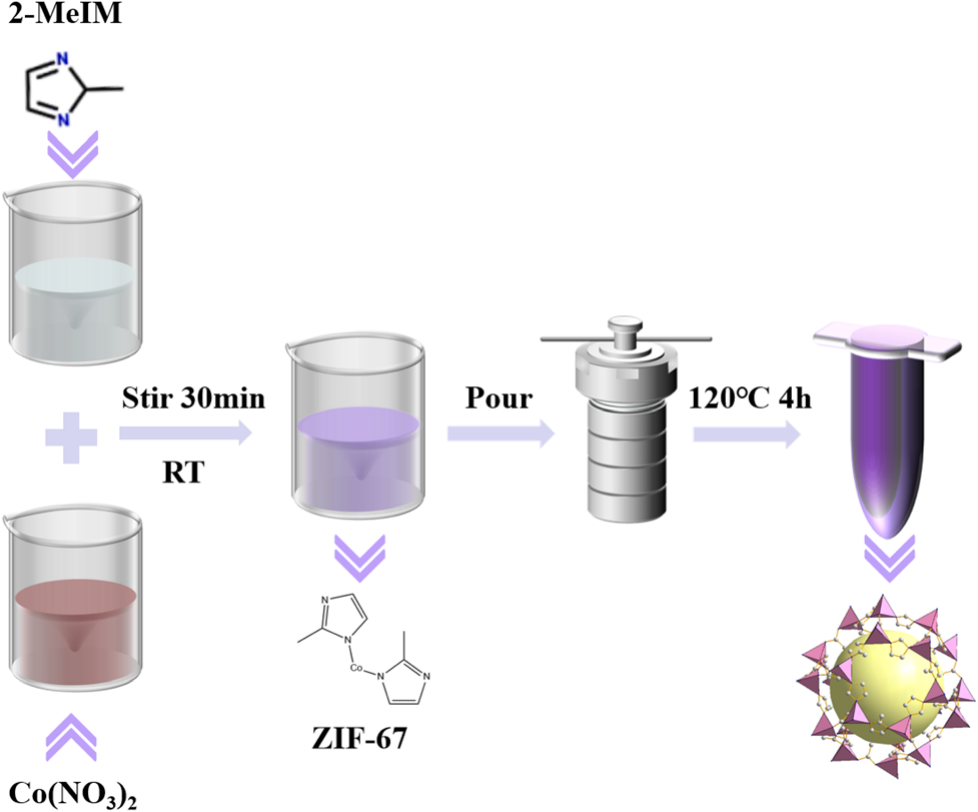
Synthetic process of ZIF-67.

As illustrated in Scheme 1, the ZIF-67 catalyst was synthesized *via* an *in situ* growth strategy. Briefly, 2-methylimidazole (2-MeIM, 0.08 mol) was dissolved in 160 mL of ethanol and stirred for 30 minutes to form precursor solution A. Concurrently, Co(NO_3_)_2_·6H_2_O (0.02 mol) was dissolved in another 160 mL of ethanol under stirring for 30 minutes to obtain precursor solution B. Subsequently, solution B was gradually added to solution A, and the mixture was stirred at room temperature for an additional 30 minutes. The resulting solution was transferred into four 100 mL stainless-steel autoclaves and heated at 120 °C for 4 hours. The ZIF-67 product was collected *via* centrifugation, washed three times with methanol to remove unreacted precursors, and finally vacuum-dried at 60 °C to yield a purple precipitate. Control samples designated as ZIF-67 (4 : 1), ZIF-67 (10 : 1), and ZIF-67 (20 : 1) were prepared by adjusting the molar ratio of 2-MeIM to Co(NO_3_)_2_·6H_2_O while maintaining other synthesis parameters constant.

### 2.3. Catalytic degradation experiments

All degradation experiments were conducted in 100 mL batch reactor containing aqueous solutions containing PMS (0.5 mM) and RhB (20 mg L^−1^), with constant magnetic stirring at 400 rpm to ensure homogeneous mixing of reactants. The initial pH of the reaction system was precisely adjusted to the target value using 0.1 M HCl or 0.1 M NaOH, and the pH was verified using a calibrated pH meter before initiating the reaction. Reactions were triggered by the addition of 20 mg of the as-prepared ZIF-67 catalyst to the pre-equilibrated PMS–RhB solution (pre-equilibrated for 10 minutes to eliminate adsorption–desorption effects between the catalyst and RhB prior to reaction initiation). At preset time intervals (*e.g.*, 10 s, 30 s, 1 min, 2 min), 1.5 mL aliquots of the reaction mixture were withdrawn using a sterile pipette, immediately mixed with 1 mL of methanol (as a radical scavenger) to rapidly terminate the reaction, and then filtered through a 0.22 µm hydrophilic polyethersulfone (PES) membrane to remove catalyst particles. The residual concentration of RhB in the supernatant was quantified using a UV-vis spectrophotometer (Shimadzu UV-2600) at its characteristic maximum absorption wavelength of 554 nm, with a blank control (methanol-aqueous solution without RhB) used to correct for background absorption. Each degradation experiment was performed in triplicate to account for experimental variability, and the average values with standard deviations were reported to ensure data reliability. Control experiments (*e.g.*, catalyst-only, PMS-only systems) were conducted under identical operating conditions to isolate the contribution of the ZIF-67/PMS synergistic effect to RhB degradation. For catalyst recyclability tests, after each degradation cycle, the used catalyst was collected *via* centrifugation at 8000 rpm for 10 minutes, thoroughly rinsed with deionized water (3 cycles of 10 mL each) to remove adsorbed RhB, intermediates, and residual PMS, and then dried in a vacuum oven at 60 °C for 4 hours to restore its structural integrity before being reused in the next cycle.

## 3. Results and discussion

### 3.1. Characterization of ZIF-67

**Fig. 1:**
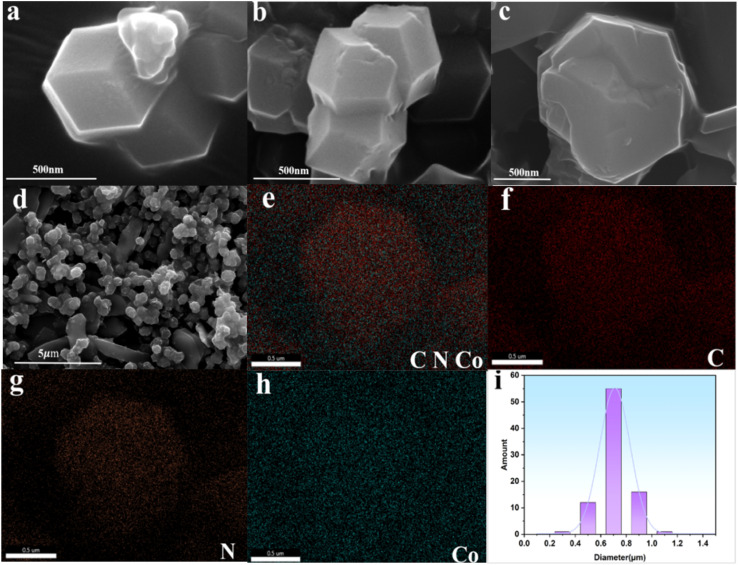
(a–d) SEM images of ZIF-67 (4 : 1). (e–h) Elemental mapping images of an individual ZIF-67 particle. (i) Size distribution histogram.

As shown in Fig. 1a–d, the introduction of excessive cobalt ions resulted in the formation of ZIF-67 particles that exhibited significant aggregation and irregular size distribution. The ZIF-67 particles possessed a regular dodecahedral morphology with an average width of around 500 nm. The corresponding elemental mapping images (Fig. 1e–h) confirmed the existence and homogeneous dispersion of Co, N, and C throughout the ZIF-67 structure.

**Fig. 2:**
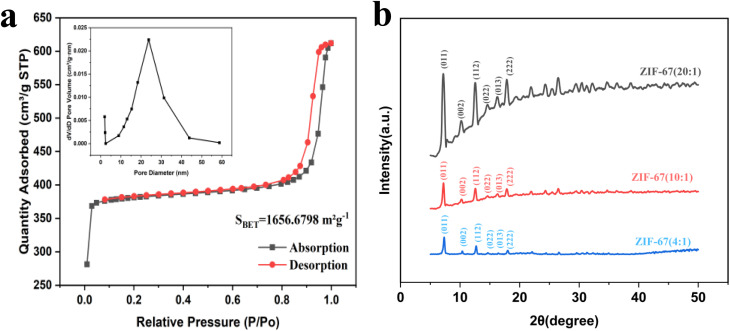
(a) N_2_ adsorption–desorption isotherm and DFT pore size distribution of ZIF-67 (4 : 1). (b) XRD pattern of ZIF-67.

The specific surface area and porosity of ZIF-67 are presented in Fig. 2a. The BET surface area of ZIF-67 was calculated as 1656.68 m^2^ g^−1^, thus confirming its highly porous nature. Pore size distribution analysis conducted using density functional theory (DFT) revealed a total pore volume of 0.832 cm^3^ g^−1^. In addition, ZIF-67 was found to have a predominantly mesoporous structure with a pore size of 25 nm. The isotherm exhibited a type-IV curve—a feature characteristic of mesoporous materials—accompanied by an H4-type hysteresis loop that is indicative of slit-like pores. The sharp increase in adsorption capacity at low relative pressure suggests the presence of abundant micropores, which is consistent with the intrinsic channels of ZIF-67 crystals.^54^

To characterize the crystal structure and phase composition of ZIF-67, XRD analysis was conducted, with the results presented in Fig. 2b. For the ZIF-67 (4 : 1) sample, the diffraction peaks appearing at 2*θ* = 7.3°, 10.4°, 12.7°, 14.7°, 16.4°, and 18.1° were espectively indexed to the (011), (002), (112), (022), (013), and (222) crystal planes of ZIF-67, respectively. Characteristic peaks of ZIF-67 were also observed in both ZIF-67 (10 : 1) and ZIF-67 (20 : 1). The intensity of the XRD characteristic peaks increased with higher 2-MeIM ratios, which can be attributed to enhanced crystal quality and phase purity. However, the highly ordered structure may also imply fewer surface defects. Meanwhile, excessive 2-MeIM could lead to complete encapsulation of metal sites by ligands, thereby resulting in a slight reduction in catalytic performance.^55,56^

### 3.2. Catalytic performance

**Fig. 3:**
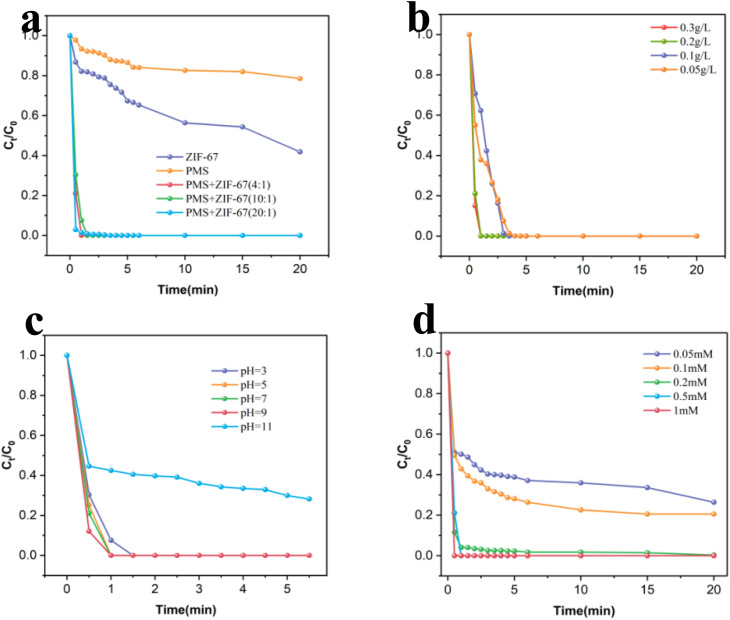
(a) Degradation efficiency of RhB by different catalysts; (b) effect of catalyst dosage; (c) effect of pH; (d) effect of PMS dosage. Reaction conditions: [catalyst] = 0.2 g L^−1^, [RhB] = 20 mg L^−1^, [PMS] = 0.5 mM, initial pH = 7.

Fig. 3a illustrates the catalytic degradation of RhB under various experimental conditions. When ZIF-67 (4 : 1) was employed without PMS, the degradation efficiency of RhB reached 58.2% within 20 minutes. In contrast, PMS alone achieved only 21.4% degradation, underscoring its negligible self-degradation capability. Remarkably, when PMS was combined with ZIF-67 catalysts prepared at different molar ratios (4 : 1, 10 : 1, and 20 : 1), complete degradation (100%) of 20 mg per L RhB was realized within 3 minutes. Among these, the ZIF-67 (4 : 1)/PMS system exhibited the most pronounced activity, achieving full degradation within a mere 1 minute. This superior efficiency highlights the suitability of ZIF-67 (4 : 1) as the optimal candidate for subsequent investigations.^57^

To refine the reaction parameters, the effects of catalyst dosage, pH, PMS concentration, pollutant concentration, and temperature were systematically evaluated.

As shown in Fig. 3b, increasing the catalyst dosage from 0.05 g L^−1^ to 0.3 g L^−1^ accelerated the degradation rate significantly, as evidenced by the progressively steeper degradation profiles. Fig. 3c presents the effect of pH. Under strongly acidic conditions (pH 3), RhB was completely degraded within 1.5 minutes. As the pH increased to 9, the degradation rate improved further, reaching completion within 90 seconds. These results confirm that the synthesized ZIF-67 demonstrates robust catalytic activity across a broad pH spectrum. However, at an initial pH of 11, the efficiency declined due to the suppression of reactive oxygen species (ROS) generation. Fig. 3d shows the influence of PMS dosage: as the PMS concentration increased from 0.05 mM to 1 mM over 20 minutes, degradation efficiency improved from 73.6% to 100%. This enhancement is attributable to the elevated ROS yield. An initial PMS concentration of 0.5 mM achieved complete degradation of 20 mg per L RhB within 60 seconds, representing an optimal balance between efficiency and economic feasibility.

**Fig. 4:**
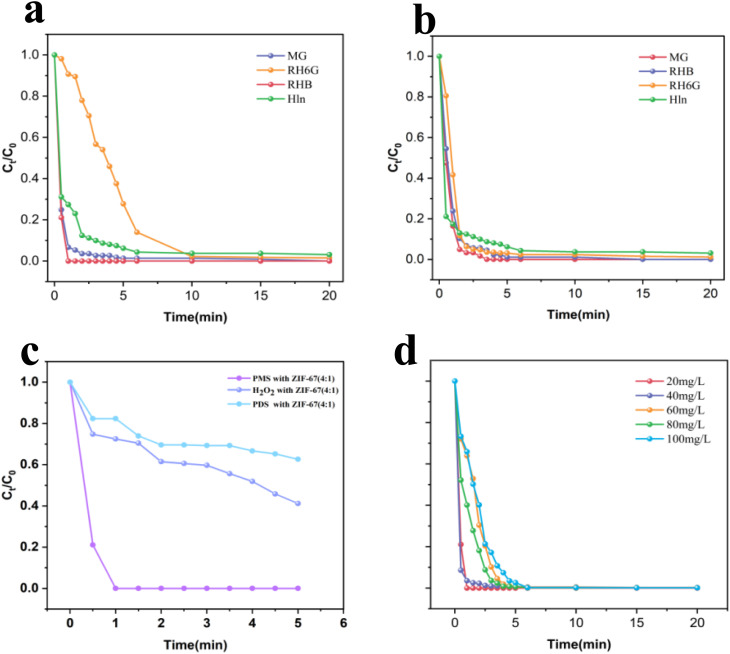
(a) Degradation efficiency towards different dyes; (b) degradation efficiency towards mixed dyes; (c) different oxidants; (d) RhB concentration. Reaction conditions: [catalyst] = 0.2 g L^−1^, [RhB] = 20 mg L^−1^, [PMS] = 0.5 mM, initial pH = 7.

Fig. 4a further compares catalytic activity toward different dyes. Within 20 minutes, degradation efficiencies followed the order: RhB (100%) = MG (100%) > RH6G (98.4%) > Hln (96.2%). In the mixed-dye system (Fig. 4b), ZIF-67 (4 : 1) maintained high activity, achieving nearly complete degradation of all dyes, which underscores its tolerance to complex matrices. Fig. 4c reveals that PMS significantly outperformed alternative oxidants (H_2_O_2_, and PDS), with complete RhB degradation occurring within 60 seconds, compared to only 58.8% and 37.3% removal by H_2_O_2_ and PDS, respectively. Fig. 4d demonstrates that even under elevated RhB concentrations, complete degradation was consistently achieved within 6 minutes, thereby confirming the catalyst's efficacy under high pollutant loads.^15,16,58,59^

### 3.3. Identification of free radicals and exploration of catalytic mechanism

**Fig. 5:**
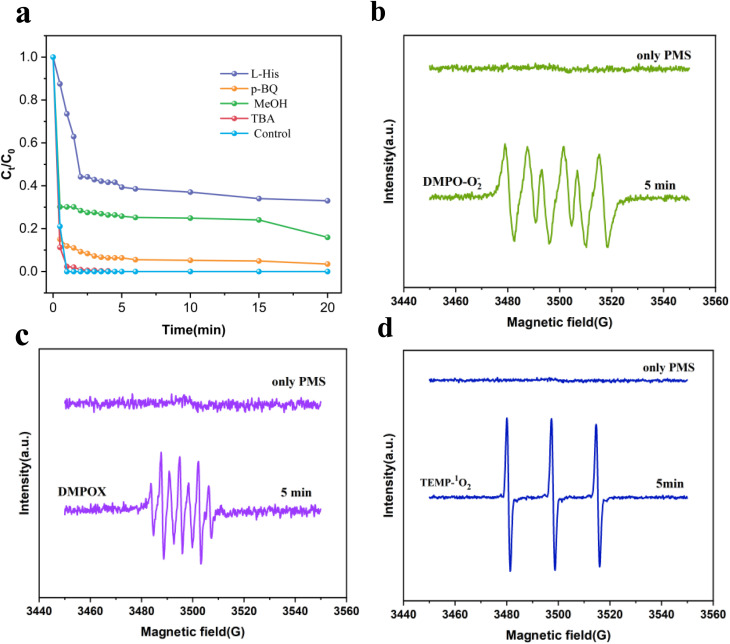
(a) Effects of scavengers (MeOH, TBA, *p*-BQ, and l-His) on RhB degradation; (b) EPR signals for DMPO-O_2_˙^−^ in the ZIF-67 (4 : 1)/PMS system; (c) DMPO-SO_4_˙^−^ and DMPO-˙OH; (d) TEMP-^1^O_2_. Reaction conditions: [catalyst] = 0.2 g L^−1^, [RhB] = 20 mg L^−1^, [PMS] = 0.5 mM, initial pH = 7.

To elucidate the role of reactive species, radical scavenging experiments were performed using l-His, MeOH, TBA, and *p*-BQ. As shown in Fig. 5a the addition of 1 mM significantly decreased RhB removal efficiency from 100% to 67%, demonstrating the crucial involvement of ^1^O_2_. MeOH (0.5 M) lowered degradation efficiency to 84%, whereas TBA (0.5 M) exerted only a marginal influence, reducing efficiency to 99.7%, indicating that SO_4_˙^−^ has a greater influence than ˙OH on RhB degradation. Similarly, the introduction of 1 mM *p*-BQ resulted in a noticeable reduction in RhB removal from 100% to 96.52%. In the initial stage after adding scavengers, both ˙OH and O_2_˙^−^ had a significant inhibitory effect on removal efficiency, which gradually diminished to negligible levels in the later phase. Given their notable suppression early in the reaction, it can be concluded that ˙OH and O_2_˙^−^ are key drivers of ROS generation during this period. Therefore, based on the results of the EPR analysis and quenching experiments described above, it can be inferred that the primary reactive species in the ZIF-67/PMS system are SO_4_˙^−^ and ^1^O_2_.

Electron Paramagnetic Resonance (EPR) was utilized to identify the reactive oxygen species (ROS) generated during PMS activation over the ZIF-67 catalyst, including SO_4_˙^−^, ˙OH, O_2_˙^−^, and singlet oxygen (^1^O_2_). The spin trapping agents 5,5-dimethyl-1-pyrroline *N*-oxide (DMPO) and 2,2,6,6-tetramethylpiperidine (TEMP) were employed to capture these radical species. DMPO effectively traps radicals such as SO_4_˙^−^, ˙OH, and O_2_˙^−^, while TEMP preferentially interacts with ^1^O_2_. As illustrated in Fig. 5b, the presence of O_2_˙^−^ in the ZIF-67 (4 : 1)/PMS system was verified, contributing to RhB degradation. According to Fig. 5c, no ESR signals corresponding to DMPO-SO_4_˙^−^ or DMPO-˙OH adducts were observed in the filtrate. Instead, seven characteristic peaks assigned to the DMPOX adduct were observed, which originated from the over-oxidation of DMPO by SO_4_˙^−^ and ˙OH.^60^ Both DMPO-SO_4_˙^−^ and DMPO-˙OH adducts are unstable and rapidly transform to DMPOX. In Fig. 5d, a clear TEMP-^1^O_2_ signal was identified in the ZIF-67 (4 : 1)/PMS system, confirming the generation of singlet oxygen.^61,62^ As demonstrated in Fig. 5c–e, no observable signals were detected in the PMS-only system, suggesting that negligible amounts of SO_4_˙^−^, ˙OH, or O_2_˙^−^ are produced by PMS alone.

**Fig. 6:**
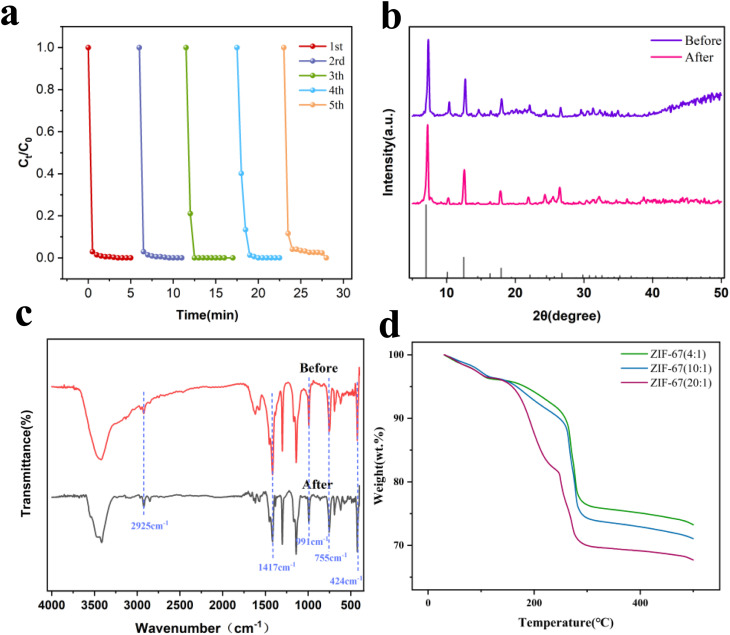
(a) Recyclability test; (b) XRD patterns; (c) FTIR spectra; (d) TGA. Reaction conditions: [catalyst] = 0.2 g L^−1^, [RhB] = 20 mg L^−1^, [PMS] = 0.5 mM, initial pH = 7.

As illustrated in Fig. 6a, the ZIF-67 catalyst retained its catalytic activity without appreciable decline after five consecutive cycles, thereby demonstrating excellent stability. It should be emphasized, however, that a slight reduction in degradation efficiency may occur, likely due to the progressive occupation of active sites by intermediate compounds adsorbed during extended reaction periods. In addition, Fig. 6b contrasts the phase structure of ZIF-67 (4 : 1) before and after the degradation reaction. After five reuse cycles, the material maintained pronounced XRD diffraction peaks consistent with those of the fresh catalyst, thereby affirming its structural stability. Furthermore, Fig. 6c displays the FTIR analysis of the catalyst before and after the reaction. ZIF-67 exhibited characteristic absorptions at 2925, 1417, 991, 755, and 424 cm^−1^. These peaks are assigned to the stretching vibrations of C–H bonds in aromatic and aliphatic moieties, the out-of-plane bending vibration of the imidazole ring, and the stretching vibration associated with Co–N bonds in the imidazolate structure, respectively.^63^ After the reaction, some characteristic peaks of 2-MeIM were partially absent. This may arise from protonation of the 2-MeIM ligand and anionic exchange, whereby the 2-MeIM ligand is substituted by anions or molecules present in the solution. Fig. 6d shows the thermogravimetric analysis of the synthesized ZIF-67 (4 : 1, 10 : 1, 20 : 1) samples. A slight weight loss (<5%) occurs between 0 and 100 °C, which is attributed to the removal of adsorbed water and solvents. A sharp weight loss is observed in the 100–300 °C range, corresponding to the decomposition of organic ligands and the collapse of the framework. Between 300 and 500 °C, the weight loss is negligible, indicating that the final residue, Co_3_O_4_, has good thermal stability.^64^

### 3.4. Toxicity analysis and degradation mechanism

Density Functional Theory (DFT) calculations were applied as a powerful tool to probe the electronic structure and catalytic activity of materials, thereby providing deep insights into the intrinsic mechanism of efficient PMS activation by ZIF-67. Electronic properties—such as the energy levels and spatial distribution of frontier molecular orbitals (HOMO and LUMO), electrostatic potential (ESP) surfaces, and intermolecular interaction energies (*e.g.* adsorption energy)—are directly linked to the material's electron-donating/accepting capability, the identification of reactive sites, and the binding strength toward PMS molecules. Collectively, these properties govern the efficiency and mechanistic pathways of reactive oxygen species (ROS) generation during PMS activation on the catalyst surface.

**Fig. 7:**
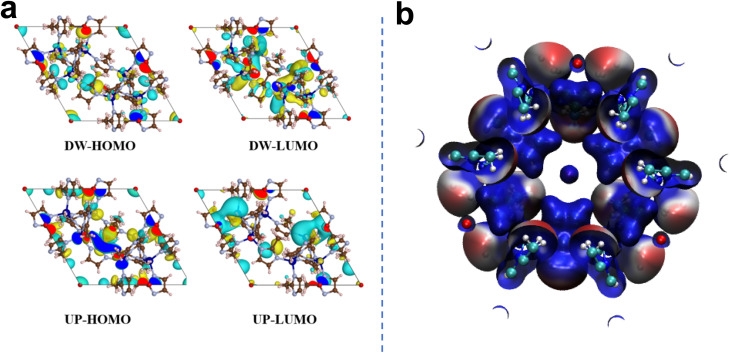
(a) Distribution of HOMO and LUMO orbitals of ZIF-67; (b) electrostatic potential (ESP) mapping.

The energy gap between the HOMO (Highest Occupied Molecular Orbital) and LUMO (Lowest Unoccupied Molecular Orbital) represents the electron transfer capability of a material. A smaller Δ*E* signifies that the material can more readily participate in redox reactions. As depicted in Fig. 7a, the HOMO–LUMO gaps for the spin-up and spin-down states of ZIF-67 were calculated to be 0.003 eV and 0.0173 eV, respectively. Such narrow gaps reflect a low energy barrier for electron transfer, which facilitates the Co^2+^/Co^3+^ redox cycle and underscores the exceptional electron migration ability of ZIF-67. This provides a theoretical basis for the redox cycling during PMS activation. Fig. 7b illustrates the electrostatic potential (ESP) map, depicting the charge distribution on the material surface. Regions with positive ESP (blue) are predisposed to adsorb anions, while regions with negative ESP (red) tend to attract cations. The ESP mapping demonstrates that the Co sites exhibit a significantly positive potential (blue regions), which promotes the adsorption of negatively charged PMS molecules on the surface, thereby furnishing abundant active sites for radical generation. Moreover, according to the ZIF-67/PMS adsorption configuration, the adsorption energy (*E*_ads_) of PMS on the ZIF-67 surface is −1.19 eV, confirming a spontaneous adsorption process. The strong adsorption ensures PMS enrichment at the catalyst interface, thereby accelerating the generation of SO_4_˙^−^. This robust interfacial interaction creates a favorable environment for PMS cleavage, yielding ^1^O_2_, ˙OH, SO_4_˙^−^, and O_2_˙^−^. These findings, together with the oxidation of Co^2+^ confirmed by XPS and the dominant radical species detected by EPR, construct a comprehensive catalytic mechanism.^65^

**Fig. 8:**
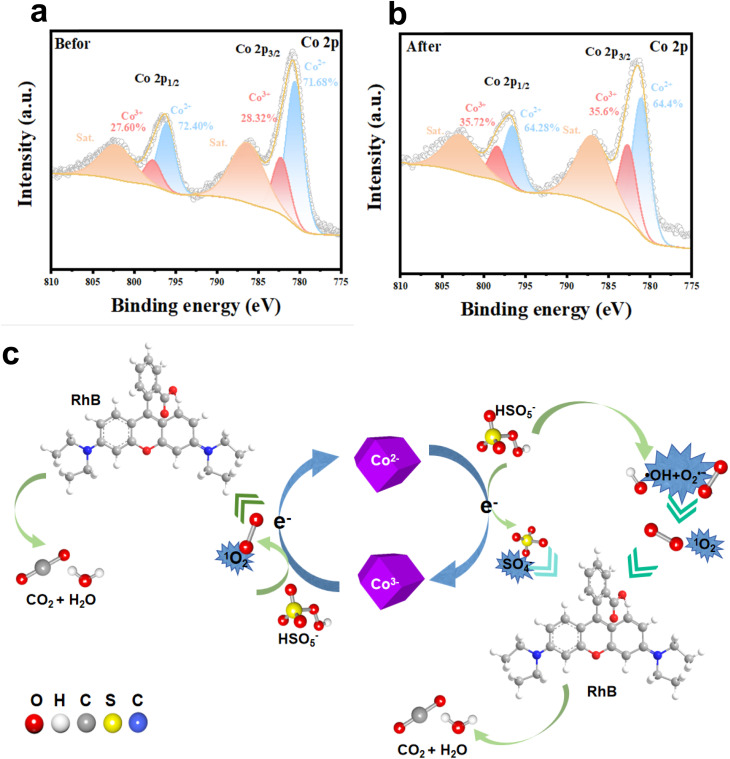
Co 2p XPS spectra of ZIF-67 (a) before and (b) after degradation. (c) Proposed reaction mechanism for pollutant degradation.

To gain a more comprehensive understanding of the charge-transfer processes occurring within the catalyst during PMS activation, a comparative analysis of the XPS spectra of the ZIF-67 sample before and after the reaction was performed. As depicted in Fig. 8a and b, the Co 2p spectrum of the catalyst was deconvoluted into six characteristic peaks. The peaks at 781.0 eV (Co 2p_3/2_) and 796.4 eV (Co 2p_1/2_) were attributed to the presence of Co^3+^, whereas the smaller peaks at 782.5 eV (Co 2p_3/2_) and 797.8 eV (Co 2p_1/2_) were assigned to Co^2+^.^66^ In addition, satellite peaks located at 786.6 eV and 802.7 eV were associated with shake-up features of Co 2p_3/2_ and Co 2p_1/2_, respectively.^67^ After the catalytic reaction, the relative proportion of Co^2+^ (Co 2p_3/2_) decreased markedly from 71.68% to 64.4%, while the fraction of Co^3+^ (Co 2p_3/2_) increased appreciably from 28.32% to 35.6%, thereby providing direct evidence for the oxidation of Co^2+^ to Co^3+^ during the degradation process.

Drawing upon theoretical calculations, XPS analysis, and ROS identification, a plausible catalytic pathway was proposed. As depicted in Fig. 8c, the mechanism for RhB degradation in the ZIF-67 catalytic system involves sequential redox cycling. Initially, Co^2+^ ions were oxidized through PMS activation (HSO_5_^−^), thereby generating ˙SO_4_^−^ radicals, concomitant with electron transfer (eqn (1)). Subsequently, Co^3+^ ions were reduced back to Co^2+^ through electron transfer from HSO_5_^−^ (eqn (2)). These reversible redox transitions sustain charge neutrality on the catalyst surface and ensure a continuous Co(ii)/Co(iii) cycle throughout PMS activation.

Following these processes, ˙OH and O_2_˙^−^ were produced *via* secondary pathways, as described in eqn (3)–(6),^68^ while the generation of ^1^O_2_ was facilitated through multi-step oxidation reactions, as represented in eqn (7)–(10).^69,70^ Importantly, the yield of ^1^O_2_ could be further enhanced by diverse oxidation pathways involving ROS, as demonstrated in eqn (11) and (12). Finally, when ˙SO_4_^−^ or ^1^O_2_ interacted with RhB molecules, intermediate compounds were formed (eqn (13)), thereby completing the degradation mechanism.

Furthermore, the generation of ^1^O_2_ can be substantially promoted through diverse oxidation processes involving ROS, as outlined in eqn (11) and (12). Ultimately, the degradation process is brought to completion when SO_4_˙^−^ or ^1^O_2_ interacts with RhB, resulting in the formation of intermediates (eqn (13)).

1 Co^2+^ + HSO_5_^−^ → Co^3+^ + SO_4_˙^−^ + OH^−^

2 Co^3+^ + HSO_5_^−^ → Co^2+^ + SO_5_˙^−^ + OH^+^

3 SO_4_˙^−^ + H_2_O → SO_4_^2−^ + ˙OH + H^+^

4 HSO_5_^−^ + H_2_O → HSO_4_^−^ + H_2_O_2_

5
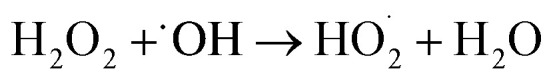
6
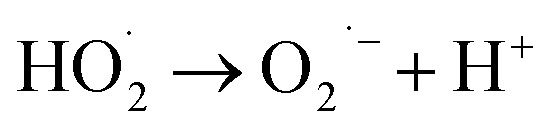

7 2O_2_˙^−^ + 2H^+^ → H_2_O_2_ + ^1^O_2_

8 O_2_˙^−^ + ˙OH → OH^−^ + ^1^O_2_

9
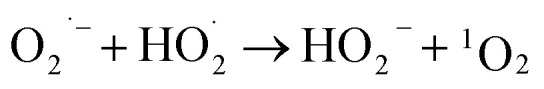

10 4˙OH → 2H_2_O + ^1^O_2_

11 2SO_5_˙^−^ + HO_2_ →1.5^1^O_2_ + 2HSO_4_^−^

12 HSO_5_^−^ + SO_5_^2−^ → ^1^O_2_ + HSO_4_^−^ + SO_4_^2−^

13 RhB + SO_4_˙^−^/^1^O_2_ → degradation products

## 4. Conclusion

This study developed a cobalt-based metal–organic framework (ZIF-67) catalyst *via* a hydrothermal method and optimized the molar ratio of 2-methylimidazole to cobalt nitrate to obtain ZIF-67 (4 : 1) with high crystallinity and a large specific surface area. The catalyst enabled rapid and complete degradation of Rhodamine B within 60 s under neutral conditions and maintained high activity over a wide pH range (3–9). Mechanistic studies confirmed that sulfate radicals and singlet oxygen are the dominant reactive species responsible for dye degradation. Furthermore, the catalyst exhibited excellent recyclability with consistent performance over five consecutive cycles. This work provides a simple and scalable synthesis strategy for efficient, stable cobalt-based catalysts with promising potential for dye wastewater treatment.

## Conflicts of interest

No potential conflict of interest was reported by the author(s).

## Data Availability

The data supporting this study are currently hosted in a private Mendeley Data repository, accessible *via* this preview link for the purpose of editorial and peer review: https://data.mendeley.com/preview/jhtfbrwdxt?a=222d2b4f-7c9d-4418-96c0-b73e20b94ba7. Upon acceptance of the manuscript, this repository will be made public to all readers.
